# Enhancement of magnon–photon–phonon entanglement in a cavity magnomechanics with coherent feedback loop

**DOI:** 10.1038/s41598-023-30693-x

**Published:** 2023-03-07

**Authors:** Mohamed Amazioug, Berihu Teklu, Muhammad Asjad

**Affiliations:** 1 LPTHE-Department of Physics, Faculty of Sciences, Ibnou Zohr University, Agadir, Morocco; 2 Department of Applied Mathematics and Sciences, Khalifa University, Abu Dhabi, 127788 United Arab Emirates; 3 Center for Cyber-Physical Systems (C2PS), Khalifa University, Abu Dhabi, 127788 United Arab Emirates

**Keywords:** Optical physics, Quantum physics

## Abstract

In this paper, we present a coherent feedback loop scheme to enhance the magnon–photon–phonon entanglement in cavity magnomechanics. We provide a proof that the steady state and dynamical state of the system form a genuine tripartite entanglement state. To quantify the entanglement in the bipartite subsystem and the genuine tripartite entanglement, we use the logarithmic negativity and the minimum residual contangle, respectively, in both the steady and dynamical regimes. We demonstrate the feasibility of our proposal by implementing it with experimentally realizable parameters to achieve the tripartite entanglement. We also show that the entanglement can be significantly improved with coherent feedback by appropriately tuning the reflective parameter of the beam splitter and that it is resistant to environmental thermalization. Our findings pave the way for enhancing entanglement in magnon–photon–phonon systems and may have potential applications in quantum information.

## Introduction

Cavity optomechanics has attracted significant attention for studying and exploiting the interaction between optical and mechanical degrees of freedom. In optomechanical systems, continuous variable (CV) states (Gaussian states) describe the information encoded in mechanical and optical modes^1–4^. In recent years cavity optomechanical system plays an essential role for studying many interesting phenomena such as quantum entangled states^5–9^. Cooling the mechanical mode to their quantum ground states^10–14^, photon blockade^15^, generating mechanical quantum superposition states^16,17^, enhancing precision measurements^18–23^, gravitational-wave detectors^24–26^ and optomechanically induced transpency^27–30^ . Quantum state transfer between separate parts is a key tool to achieve quantum information processing protocols and quantum communications^31–33^. Recently, cavity magnomechanics offers a robust platform where ferrimagnetic cristal (e.g., yttrium iron garnet (YIG) sphere) is coupled with a microwave cavity^38–40^. We note that the Kittel mode^41^ in the YIG sphere can realize strong coupling with the microwave photons in a high-quality cavity, leading to cavity polaritons^42–46^ and the vacuum Rabi splitting. Also, in the cavity magnomechanics, a magnon mode (spin wave) is combined with a vibratory deformation mode of a ferromagnet (or ferrimagnet) by the magnetostrictive force, and a microwave cavity mode by the interaction of magnetic dipoles. The magnetostrictive interaction is a dispersive interaction similar to a radiation pressure for a large ferromagnet, where the frequency of the mechanical mode is very lower than the magnon frequency^47,48^. Besides, the first realization of the magnon–photon–phonon interaction^47^.

Entanglement can be viewed as a key resource for quantum information processing. This concept was introduced by Schrödinger in his replay to the EPR-paradox proposed by Einstein et al.^49,50^. It enables quantum communication protocols such as quantum teleportation^51^, superdense coding^52^, telecloning^53^ and quantum cryptography^54^. The logarithmic negativity^55–57^ measure the amount of bipartite entanglement systems characterized by continuous variables (CV) of Gaussian states. In this work we consider the coherent feedback loop to improve of entanglement in optomagnomechanical system. This technique is studied theoretically in optomechanical systems^58–60^ and recently realized experimentally in optomechanical systems^34–37^.

In this work, we investigate theoretically the improvement of entanglement of three bipartites systems and tripartite Gaussian states in an optomagnomechanical system composed of a Fabry-Perot cavity content inside YIG sphere via coherent feedback. A microwave field (not shown) is implemented to improve magnon-phonon coupling. At YIG sphere site, the magnetic field (along x axis) of the cavity mode, the drive magnetic field (in y direction), and biased magnetic field (z direction) are common perpendicular. The coherent feedback technique is presently implemented experimentally in optomechanical systems^34–37^. We employ the logarithmic negativity to quantify the quantum correlations of three bipartite mode and genuine tripartite entanglement state in stationary-state and in dynamical state. We discuss the evolution of the entanglement of each bipartite Gaussian states and genuine tripartite entanglement state under the effect of the temperature. We demonstrate the role of the feedback technique to make the entanglement robust under the variation of physical parameters characterizing the optomagnomechanical system. We first demonstrate that the genuine tripartite magnon-phonon-photon entanglement exists in the system, if the magnon mode is in resonance with anti-Stokes (blue sideband) and the cavity mode is in resonance with Stokes (red sideband)^61^. Magnon squeezing enhanced ground-state cooling in cavity magnomechanics^62^. Entanglement enhancement in cavity magnomechanics by an optical parametric amplifier^63^. In this paper, we consider the effects of coherent feedback loop on tripartite entanglement. In a linearized description, the optomechanical system can be viewed as analogy to a linear amplifier^64^ that receives both optical and mechanical responses to quantum and classical fluctuations.

The paper is structured as follows. The Hamiltonian and the corresponding non linear quantum Langevin equations of the opto-magno-mechanical of the system are introduced in “The Model” section. In “Linearization of quantum Langevin equations” section, using the linear quantum Langevin equation, we derive the covariance matrix of the tripartite system. In “Quantification of entanglement” section, we employ the logarithmic negativity to derive the entanglement of three bipartite modes and tripartite mode. The results and discussions are given in “Results and discussion” section.

**Figure 1:**
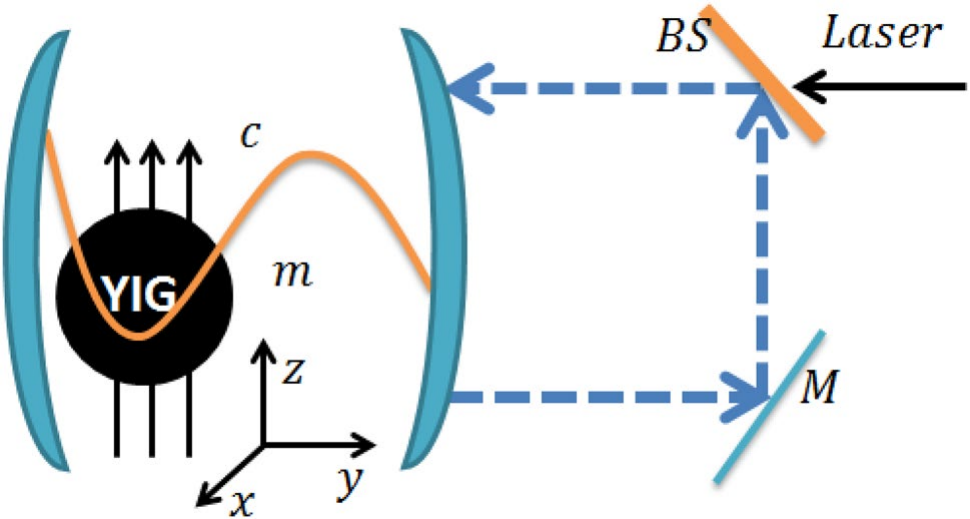
Schematic diagram of a single-mode cavity with feedback loop and a YIG sphere. The cavity is also driven by an electromagnetic field with amplitude $\Omega$, an input light field enters in the cavity across an asymmetric beam splitter (BS). The output field is totally reflected on the mirror *M* and a part of the output field is sent to the cavity via beam splitter. The magnons are embodied by a collective motion of a large number of spins in a macroscopic ferrimagnet, and the magnon mode is directly driven by a microwave source (not shown) to enhance the magnomechanical coupling. The cavity photons and magnons are coupled via magnetic dipole interaction, and the magnons and phonons are coupled via magnetostrictive (radiation pressure-like) interaction.

## The model

The system which we consider consists of a cavity magnomechanics driven by single coherent laser source and the microwave. A YIG sphere (a 250-$\mu \hbox {m}$-diameter sphere) is used as in Ref. ^47^ is placed inside the cavity, and where the coherent feedback loop is implemented as shown in Fig. 1. The magnetic dipole interaction mediates the coupling between magnons and cavity photons. The magnons are coupled with phonons through magnetostrictive interaction. The variable magnetization induced by the magnon excitation inside the YIG sphere results in the deformation of its geometrical structure, which forms vibrational modes (phonons) of the sphere, and vice versa ^65^. We consider the size of the sphere is much smaller than that of the microwave wavelength, as a result the influence of the radiation pressure is negligible. The Hamiltonian of the system is given by

1 $$\begin{aligned} {\mathcal{H}} = {\mathcal{H}}_{free} + {\mathcal{H}}_{md} + {\mathcal{H}}_{mc} + {\mathcal{H}}_{dm} + {\mathcal{H}}_{dc}. \end{aligned}$$

The first term of ${\mathcal{H}}$ describes the free magnon and optical modes which can be written as

2 $$\begin{aligned} {\mathcal{H}}_{free} = \hbar \omega _c c^{\dag } c + \hbar \omega _m m^{\dag } m + \frac{\hbar \omega _d}{2} ( q^2 + p^2 ), \end{aligned}$$

where *c* ($c^{\dag }$) and *m* ($m^{\dag }$) ($[O, O^{\dag }]\,{=}\,1$, $O\,{=}\,c,m$) are the annihilation (creation) operator of the cavity and magnon modes, respectively, *q* and *p* ($[q, p]\,{=}\,i$) are the dimensionless position and momentum quadratures of the mechanical mode, and $\omega _c$, $\omega _m$, and $\omega _d$ are respectively the resonance frequency of the cavity, magnon and mechanical modes. The magnon frequency is determined by the external bias magnetic field *H* and the gyromagnetic ratio $\gamma$, i.e., $\omega _m=\gamma H$. The second term in Eq. (1) is the Hamiltonian describing the interaction between magnon and mechanical modes described by

3 $$\begin{aligned} {\mathcal{H}}_{md} = \hbar g_{md} m^{\dag } m q. \end{aligned}$$

The single-magnon magnomechanical coupling rate $g_{md}$ is small, but the magnomechanical interaction can be improved via driving the magnon mode with a strong microwave field (directly driving the YIG sphere with a microwave source^66,67^). The third term in Eq. (1) gives the interaction between the optical field and the magnon. It reads as

4 $$\begin{aligned} {\mathcal{H}}_{mc} = \hbar g_{mc} (c + c^{\dag }) (m + m^{\dag }). \end{aligned}$$

The coupling rate $g_{mc}$ between the magnon and microwave can be larger than the dissipation rates $\kappa _c$ and $\kappa _m$ of the cavity and magnon modes respectively, entering into the strong coupling regime, $g_{mc} > \kappa _{c}, \kappa _{m}$ ^42–46^. In the rotating wave approximation (RWA), the expression, $g_{mc} (c + c^{\dag }) (m + m^{\dag }) \rightarrow g_{mc} (c m^{\dag } + c^{\dag } m)$ (valid when $\omega _c, \omega _m \gg g_{mc}, \kappa _{c}, \kappa _{m}$, which is easily satisfied ^47^). The fourth term in the Hamiltonian (1) represent the magnon mode is directly driven by a microwave source (not shown) to enhance the magnomechanical coupling. It is given by

5 $$\begin{aligned} {\mathcal{H}}_{dm} = i \hbar {{\mathcal{E}}} (m^{\dag } e^{-i \omega _0 t} - m e^{i \omega _0 t} ), \end{aligned}$$

where ${{\mathcal{E}}} =\frac{\sqrt{5}}{4} \gamma \! \sqrt{N} B_0$ is the Rabi frequency  ^61^ describes the coupling strength of the drive magnetic field (with $B_0$ and $\omega _0$ are respectively the amplitude and frequency ) with the magnon mode, where $\gamma /2\pi = 28$ GHz/T, and the total number of spins $N=\rho V$ with *V* the volume of the sphere and $\rho =4.22 \times 10^{27}$ m$^{-3}$ the spin density of the YIG. YIG sphere is fixed at the antinode of the magnetic field of the cavity mode. The Rabi frequency ${{\mathcal{E}}}$ is derived under the hypothesis of the low-lying excitations, $\langle m^{\dag } m \rangle \ll 2Ns$, with $s=\frac{5}{2}$ is the spin number of the ground state Fe$^{3+}$ ion in YIG. The last term in the Hamiltonian (1) characterize the optical field derived of the system which is transmitted by the beam splitter. It is given by

6 $$\begin{aligned} {\mathcal{H}}_{dc} = \hbar \Omega \mu (c^\dagger e^{i \phi } + c e^{-i \phi } ), \end{aligned}$$

where $\phi$ is the phase of electromagnetic field, the quantity $\mu$ and $\tau$ denote the real amplitude transmission parameters of the beam splitter satisfies the equation $\mu ^2 + \tau ^2 = 1$ ($\mu$ and $\tau$ are real and positive)^60^. The quantum Langevin equations (QLEs) characterizing the system are given by

7 $$\begin{aligned} 
\dot{c}= & {} - (i \Delta _{fb} + \kappa _{fb}) c - i g_{mc} m -i \mu \Omega e^{i \phi } + \sqrt{2 \kappa _c} c_{fb}^{\textrm{in}}, \nonumber \\ \dot{m}= & {} - (i \delta _m + \kappa _m) m - i g_{mc} c - i g_{md} m q + {{\mathcal{E}}} + \sqrt{2 \kappa _m} m^{\textrm{in}}, \nonumber \\ \dot{q}= & {} \omega _d p, \nonumber \\ \dot{p}= & {} - \omega _d q - \gamma _d p - g_{md} m^{\dag }m + \chi , \end{aligned}$$

where $\kappa _{fb}=\kappa _c(1-2\tau \cos {\theta })$ and $\Delta _{fb}=\Delta _{c}-2\kappa _c\tau \sin {\theta }$ (with $\Delta _{c}=\omega _{c}-\omega _0$) are respectively the effective cavity decay rate and the detuning with $\theta$ describes the phase shift generated by the reflectivity of the output field on the mirrors. The operator $C^{in}_{fb}=\tau {e}^{{i}\theta }c^{out} +\mu c^{in}$ describes the input optical field induced via the coherent feedback technique. Besides, the output field $c^{out}$ and the cavity field *c* are related via standard input-output relation $c^{out} = \sqrt{2\kappa _c}c - \mu c^{in}$^68^ (i.e. $C^{in}_{fb}=\tau \sqrt{2\kappa _c}{e}^{{i}\theta }c+c^{in}_{fb}$). In addition, the non-zero coherent feedback correlations properties of the input noise operators for the cavity $c^{in}_{fb}$ and $c^{in+}_{fb}$ (where $c^{in}_{fb}=\mu (1-\tau {e}^{{i}\theta })c^{in}$)^69^, are given by

8 $$\begin{aligned} \langle c_{fb}^{\textrm{in}}(t) \, c_{fb}^\mathrm{in \dag }(t')\rangle= & {} \mu ^2 (1-\tau {e}^{{i}\theta })(1-\tau {e}^{-{i}\theta }) [n_c(\omega _c){+}1] \,\delta (t{-}t'), \nonumber \\ \langle c_{fb}^\mathrm{in \dag }(t) \, c_{fb}^{\textrm{in}}(t')\rangle= & {} \mu ^2 (1-\tau {e}^{{i}\theta })(1-\tau {e}^{-{i}\theta }) n_c(\omega _c) \, \delta (t{-}t') \end{aligned}$$

with $\delta _{m}=\omega _{m}-\omega _0$, $\kappa _m$ is the dissipation rate of the magnon mode, $\gamma _d$ is the mechanical damping rate, and $m^{\textrm{in}}$ and $\xi$ are input noise operators for the magnon and mechanical modes, respectively, which are zero mean and characterized by the following correlation functions ^69^

9 $$\begin{aligned} \langle m^{\textrm{in}}(t) \, m^\mathrm{in \dag }(t')\rangle= & {} [n_m(\omega _m)+1] \, \delta (t{-}t'),\nonumber \\ \langle m^\mathrm{in \dag }(t) \, m^{\textrm{in}}(t')\rangle= & {} n_m(\omega _m)\, \delta (t{-}t'),\nonumber \\ \langle \chi (t)\chi (t')\,{+}\,\chi (t') \chi (t) \rangle /2 {\simeq}& {} \gamma _d [2 n_d(\omega _d) {+}1] \delta (t{-}t'), \end{aligned}$$

where $n_j(\omega _j){=}\big [ \textrm{exp}\big ( \frac{\hbar \omega _j}{k_B T} \big ) {-}1 \big ]^{-1}$ $(j{=}c,m,d)$ are the equilibrium mean thermal photon, magnon, and phonon number, respectively. The mechanical quality factor ${\mathcal{Q}} = \omega _d/\gamma _d \,\, {\gg }\, 1$ is large for a Markovian approximation^70^.

## Linearization of quantum Langevin equations

The analytical solution of quantum Langevin equations, Eqs. (7), can be obtain by using the following linearization scheme $O=O_s +\delta O$ ($O\, {=}\, c,m,q,p$), i.e. we decompose the mode operators as a sum of the steady state average and a fluctuation quantum operator and neglecting second order fluctuation terms when the magnon mode is strongly driven (large amplitude $|m_s| \gg 1$ at the steady state), and the cavity field also has a large amplitude $|c_s| \gg 1$ via the cavity-magnon beamsplitter interaction. This allows us to linearize the dynamics of the system around the steady-state values as

10 $$\begin{aligned} c_s=-\frac{ig_{mc}m_s+i\mu \Omega {e}^{i\phi }}{i\Delta _{fb}+\kappa _{fb}}, \qquad m_s = \frac{ -ig_{mc}c_s + {{\mathcal{E}}} }{ i \Delta _m + \kappa _m } \end{aligned}$$

and for $|\Delta _m|, |\Delta _{fb}| \gg \kappa _{fb}, \kappa _m$, the steady-state value of the magnon mode $m_s$ can be written as

11 $$\begin{aligned} m_s \simeq \frac{ i {{\mathcal{E}}} \Delta _{fb} -i\mu \Omega {e}^{i\phi }}{g_{ma}^2 - \Delta _m \Delta _{fb} }, \end{aligned}$$

where $\Delta _m = \delta _m + g_{md} q_s$ is the effective magnon-drive detuning including the frequency shift due to the magnomechanical interaction, and $G_{md} = i \sqrt{2} g_{md} m_s$ is the effective magnomechanical coupling rate, where $q_s = - \frac{g_{md}}{\omega _d} m_s^2$.

The linearized QLEs describing the quadrature fluctuations $(\delta Q, \delta P, \delta x, \delta y, \delta q, \delta p)$, with $\delta Q=(\delta c + \delta c^{\dag })/\sqrt{2}$, $\delta P=i(\delta c^{\dag } - \delta c)/\sqrt{2}$, $\delta x=(\delta m + \delta m^{\dag })/\sqrt{2}$, and $\delta y=i(\delta m^{\dag } - \delta m)/\sqrt{2}$, is given by

12 $$\begin{aligned} \dot{\Lambda } (t) = {{\mathcal{F}}} \Lambda (t) + \nu (t) , \end{aligned}$$

where $\Lambda (t)=\big [\delta Q (t), \delta P (t), \delta x (t), \delta y (t), \delta q (t), \delta p (t) \big ]^T$ is the vector of the quadrature fluctuations, $\nu (t) = [ \sqrt{2\kappa _c} Q^{\textrm{in}} (t), \sqrt{2\kappa _c} P^{\textrm{in}} (t), \sqrt{2\kappa _m} x^{\textrm{in}} (t), \sqrt{2\kappa _m} y^{\textrm{in}} (t), 0, \chi (t) ]^T$ is the vector of input noises, and the drift matrix ${{\mathcal{F}}}$ can be written as

13 $$\begin{aligned} {{\mathcal{F}}} = \begin{pmatrix} -\kappa _{fb} &{} \Delta _{fb} &{} 0 &{} g_{mc} &{} 0 &{} 0 \\ -\Delta _{fb} &{} -\kappa _{fb} &{} -g_{mc} &{} 0 &{} 0 &{} 0 \\ 0 &{} g_{mc} &{} -\kappa _m &{} \Delta _m &{} -G_{md} &{} 0 \\ -g_{mc} &{} 0 &{} -\Delta _m &{} -\kappa _m &{} 0 &{} 0 \\ 0 &{} 0 &{} 0 &{} 0 &{} 0 &{} \omega _d \\ 0 &{} 0 &{} 0 &{} G_{md} &{} -\omega _d &{} -\gamma _d \\ \end{pmatrix}. \end{aligned}$$

The drift matrix in Eq. (13) is provided under the condition $| \Delta _m|, |\Delta _{fb}| \gg \kappa _{fb}, \kappa _m$. In fact, we will show later that $| \Delta _m|, |\Delta _{fb}| \simeq \omega _d \gg \kappa _{fb}, \kappa _m$ are optimal for the presence of all bipartite entanglements of the system. Note that Eq. (10) is intrinsically nonlinear since $\Delta _m$ contains $|m_s|^2$. However, for a given value of $\Delta _m$ (one can always alter $\Delta _m$ by adjusting the bias magnetic field) $m_s$, and thus $G_{md}$, can be achieved straightforwardly.

The time evolution of the quantum fluctuations of the system is a continuous variable (CV) three-mode Gaussian state is completely characterized by a $6\times 6$ covariance matrix (CM) $\Gamma$, where $\Gamma _{ij}=\frac{1}{2}\langle \Lambda _i(t) \Lambda _j(t') + \Lambda _j(t') \Lambda _i(t) \rangle$ ($i,j=1,2,...,6$) of the covariance matrix (CM) $\Gamma$ satisfies^1,71^

14 $$\begin{aligned} d\Gamma /dt = {{\mathcal{F}}} \Gamma + \Gamma {{\mathcal{F}}}^T + {\mathcal{D}}, \end{aligned}$$

where ${\mathcal{D}}=\textrm{diag} \big [ \kappa _c \mu ^2 (1-\tau )^2 (2n_c+1), \kappa _c\mu ^2 (1-\tau )^2 (2n_c+1), \kappa _m (2n_m+1), \kappa _m (2n_m+1), 0, \gamma _d (2n_d +1 ) \big ]$ is the diffusion matrix, which is defined through $\langle \nu _i(t) \nu _j(t') + \nu _j(t') \nu _i(t) \rangle /2 = {\mathcal{D}}_{ij} \delta (t-t')$ and $\Gamma _0 = \textrm{diag}(1,1,1,1,1,1)$ is the CM of the tripartite system at $t=0$.

## Quantification of entanglement

Entanglement is a key resource for various quantum information technologies, so quantification of entanglement is an important problem. Entanglement of formation is a proper way to quantify entanglement, but an analytical expression for this measure exists only for special cases. In this work, we investigate the entanglement of a two-mode CV system may be quantified by different entanglement monotones, entanglement negativity^55–57^ and entanglement of formation^72–75^. Both may be computed starting from the covariance matrix of the system. Here, for analytic convenience, we adopt the negativity $E_N$ as an entanglement quantifier,

15 $$\begin{aligned} {{\mathcal{E}}}_N = \max [0,-\log (2\xi ^-)] \end{aligned}$$

with $\xi ^-$ being the smallest symplectic eigenvalue of partial transposed covariance matrix of two mode Gaussian states

16 $$\begin{aligned} \xi ^-= \sqrt{\frac{\sigma -\sqrt{\sigma ^2-4\det \Gamma }}{2}}. \end{aligned}$$

The covariance matrix $\Gamma$ associated with the two magnon modes is given by

17 $$\begin{aligned} \Gamma = \begin{pmatrix} {{\mathcal{A}}} &{} {\mathcal{C}} \\ {\mathcal{C}}^T &{} {{\mathcal{B}}} \end{pmatrix}. \end{aligned}$$

The $2\times 2$ sub-matrices ${{\mathcal{A}}}$ and ${{\mathcal{B}}}$ in Eq. (17) describe the autocorrelations of the two magnon modes and $2\times 2$ sub-matrix ${\mathcal{C}}$ in Eq. (17) denotes the cross-correlations of the two magnon modes. The symbol $\Gamma$ is written as $\Gamma =\det {{\mathcal{A}}}+\det {{\mathcal{B}}}-\det {\mathcal{C}}$. The two subsystems are entangled if ${{\mathcal{E}}}_N>0$.

To investigate tripartite entanglement of the system, we use the residual contangle ${\mathcal{R}}$ ^76^ as quantitative measure, where contangle is a CV analogue of tangle for discrete-variable tripartite entanglement ^77^. A *bona fide* quantification of tripartite entanglement is given by the *minimum* residual contangle ^76^

18 $$\begin{aligned} {\mathcal{R}}_{\textrm{min}} \equiv \textrm{min} \Big [ {\mathcal{R}}^{c|md}, \, {\mathcal{R}}^{m|cd}, \, {\mathcal{R}}^{d|cm} \Big ] \end{aligned}$$

with ${\mathcal{R}}^{i|jk} \equiv C_{i|jk} - C_{i|j} - C_{i|k} \ge 0$ ($i,j,k=c,m,d$) is the residual contangle, with $C_{v|w}$ the contangle of subsystems of *v* and *w* (*w* contains one or two modes), which is a proper entanglement monotone defined as the squared logarithmic negativity^76^. Besides, a nonzero minimum residual contangle ${\mathcal{R}}_{\textrm{min}}\,>\,0$ exhibit the existence of *genuine* tripartite entanglement in the system. ${\mathcal{R}}^{i|jk}>0$ is similar to the Coffman-Kundu-Wootters monogamy inequality ^77^ hold for the system of three qubits.

## Results and discussion

In this section, we will discuss the steady state quantum correlations of two magnon under different effects by considering experimental values reported in^61^: $\omega _{c}/2\pi = 10$ GHz, $\omega _{d}/2\pi = 10$ MHz, $\gamma _d/2\pi = 100$ Hz, $\kappa _{c}/2\pi = \kappa _{m}/2\pi = 1$ MHz, $g_{mc}/2\pi = G_{md}/2\pi =3.2$ MHz, and at low temperature $T = 10$ mK. $G_{md} = 2\pi \times 3.2$ MHz implies the drive magnetic field $B_0 \approx 3.9\times 10^{-5}$ T for $g_{md}\approx 2\pi \times 0.2$ Hz, corresponding to the drive power $P =8.9$ mW.

**Figure 2:**
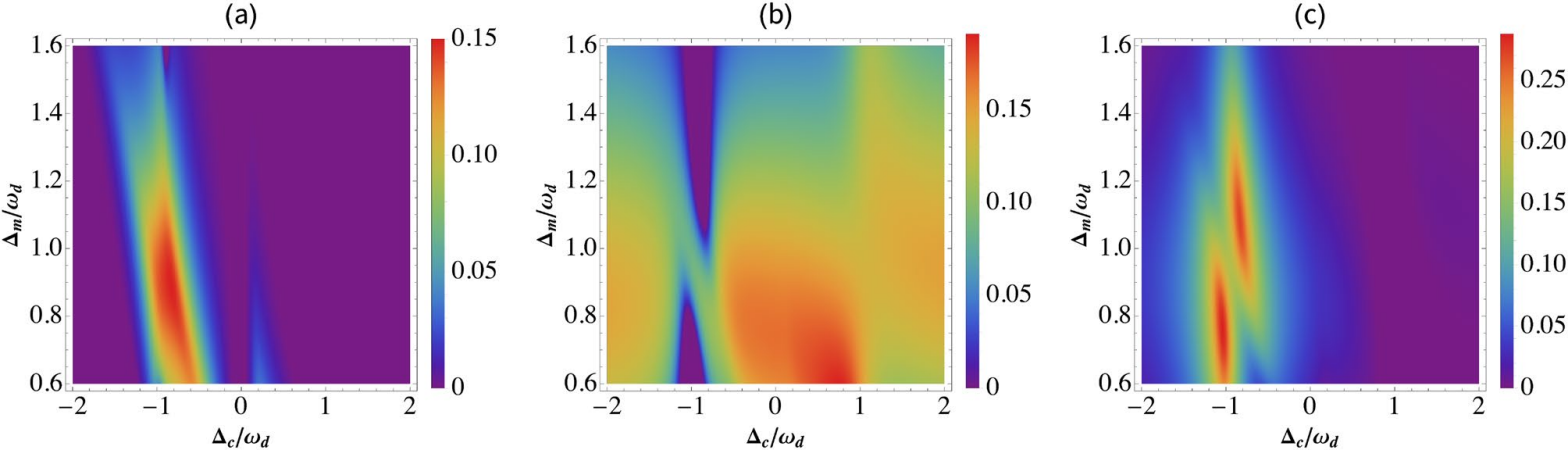
Plot of bipartite entanglement (**a**) *Eom*, (**b**) *EmM*, and (**c**) *EoM* versus detunings $\Delta _c$ and $\Delta _m$ with $\tau =0.1$ and $\theta =0$. See text for the other parameters.

In Fig. 2, we plot three bipartite entanglement *Eom* (between the cavity and magnon mode), *EmM* (between the magnon and mechanical mode) and *EoM* (between the cavity and mechanical mode) in steady state as a function of the detunings $\Delta _c$ and $\Delta _m$ in the presence of coherent feedback loop . We remark, the maximum value of entanglement of the three bipartite is enhanced by coherent feedback loop in comparison with results in Ref.^61^. This can explain by the re-injection of the photon in the cavity which improves the coupling between different bipartite modes. We observe, when $\Delta _c = - \Delta _d$ the entanglement *Eom* and *EoM* is maximum while the entanglement *EmM* is 0.10.

**Figure 3:**
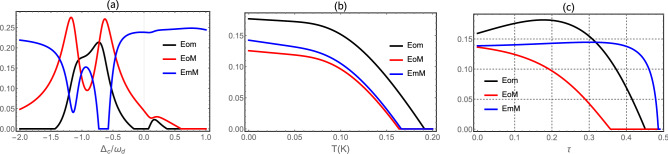
(**a**) Plot of *Eom*, *EoM* and *EmM* as a function of $\Delta _c/\omega _d$, (**b**) temperature and (**c**) reflectivity $\tau$. We take $G_{md}/2\pi = 4.8$ MHz and $\Delta _m = 0.9 \omega _d$. The other parameters are same as in Fig. 2;$\tau =0.1$ (**a**,**b**) $\Delta _c = - \omega _d$ and $n_d = 20.34$ ($T=10$ mK) (**a**–**c**). See text for the other parameters.

In Fig. 3, we present the steady state of the three bipartite entanglements *Eom*, *EoM* and *EmM* versus different parameters. The three bipartite entanglements are all not vanishing in different regions of $\Delta _c/\omega _d$ in Fig. 3a. This means the presence of tripartite entanglement between photon-magnon-phonon. We remark that the three bipartite entanglement are robust against temperature and survive up to about 200 mK (see Fig. 3b) as also discussed in Ref.^61^. We can explain the diminishing of all bipartite entanglement by the thermal effects induced by decoherence phenomenon^78^. Besides, the two bipartite entanglement *Eom* and *EoM* are enhanced with increasing values of the reflectity parameters $\tau$ (i.e. decay rate $\kappa _{fb}$ reduces) and begin to decrease quickly after reach its maximum entanglement value, i.e. one can say that the coherent feedback enhances the bipartite entanglement as in Fig. 3c. Moreover, the entanglement between magnon and phonon is decreases quickly with increasing $\tau$. This can be explained by the decoherence effects produce with the re-injection of photons in the cavity, because increasing the photon number is responsible of more thermal effects which induce the degradation of the quantum correlations between the two modes as also discussed in Ref.^60^.

**Figure 4:**
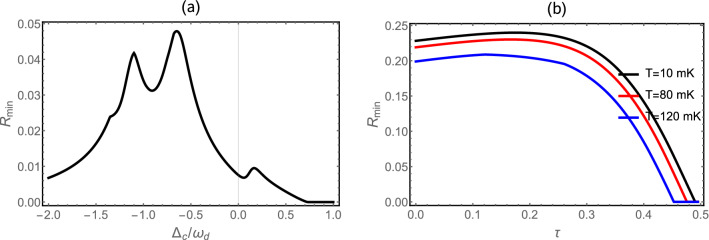
(**a**) Plot of tripartite entanglement in terms of the minimum residual contangle $R_{min}$ versus normalized detuning $\Delta _c/\omega _d$ with $n_d = 20.34$ ($T=10$ mK). (**b**) The residual contangle $R_{min}$ versus the reflectivity $\tau$ for different values of the temperature $T=10$ mK (black), $T=80$ mK (Red) and $T=120$ mK (Blue). We take $G_{md}/2\pi = 4.8$ MHz, $\Delta _m = 0.9 \omega _d$, $\Delta _c = - \omega _d$ and $\theta =0$. See text for the other parameters.

We plot in Fig. 4a we plot in steady state the minimum of the residual contangle $R_{min}$ versus of detuning $\Delta _c/\omega _d$ with $G_{md}/2\pi = 4.8$ MHz as in Ref.^61^, for a fixed value of all other parameters. We notice that the system is a genuinely tripartite entangled state as shown by the nonzero minimum residual contangle $R_{min}$ in Fig. 4b. Also we plot the evolution of the minimum of the residual contangle versus the reflectivity $\tau$ for different values of the temperature *T* as implemented in Fig. 4b. Firstly, we remark the enhancement of tripartite entanglement with increasing the parameter $\tau$, i.e., the coherent feedback loop enhances tripartite entanglement. This tripartite entanglement is decreases quickly after reaching its maximum value for a specific value of $\tau$. Besides, the $R_{min}$ decreases with increasing the temperature (decoherence phenomenon), i.e. a higher temperature reduces the amount of the tripartite entanglement. Also the region in which tripartite entanglement exists increases with decreasing temperature as shown in Fig. 4b.

**Figure 5:**
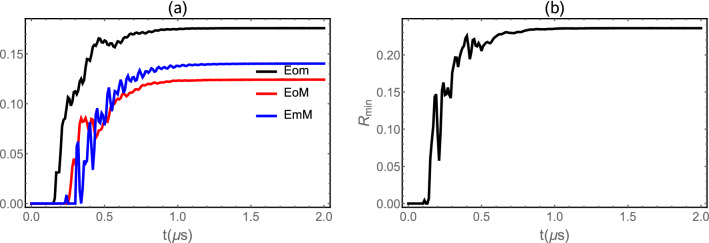
(**a**) Time evolution of the all bipartite entanglement *Eom*, *EoM* and *EmM* with $G_{md}/2\pi = 4.8$ MHz, $\tau =0.1$, $\Delta _c =-\omega _d$, $n_d = 20.34$ ($T=10$ mK) and $\theta =0$. (**b**) Plot of contangle $R_{min}$ as function of time $t(\mu s)$. See text for the other parameters.

We plot in Fig. 5a time-evolution of the three bipartite entanglement *Eom*, *EoM* and *EmM*. We remark that the entanglement in the Fig. 5a exhibits three regimes of the entanglement of the all three entanglement. The first regime concerns classically correlated states (zero entanglement), i.e. when two are separated. This indicates the absence of any quantum correlations transfer between two modes. The second regime corresponds to the emergence of entanglement between the two modes. In this regime we observe the generation of the oscillation in time this can be explained by the Sørensen-Mølmer entanglement dynamics discussed in Ref.^58^. The third regime, corresponds to large periods of evolution and associated with the entanglement between the two modes when they reach the steady regime. We remark in Fig. 5b that the system is a genuinely tripartite entangled state as shown by the nonzero minimum residual contangle $R_{min}$.

## Conclusions

In summary, we have proposed a theoretical scheme to enhance the three bipartite and tripartite entanglement in optomagnomechanical system. We have quantified the amount of entanglement in all bipartite and tripartite mode via logarithmic negativity. We have shown the genuinely tripartite entanglement state via the nonzero minimum residual contangle ${\mathcal{R}}_{min}$. Furthermore, we have discussed the behavior of stationary and dynamics of three bipartite and tripartite entanglement versus the parameter reflective of beam splitter and the phenomenon of decoherence effects using experimentally feasible parameters. We have shown that the presence of coherent feedback loop enhances the bipartite photon-magnon and magnon-phonon entanglement *Eom* and *EmM*, respectively. Moreover, the presence of coherent feedback loop degrade the photon-phonon entanglement *EoM* as shown in Fig. 3c. Our results show that the entanglement is fragile under thermal (decoherence) effects while the robustness of entanglement in the presence of coherent feedback can be achieved.

## Data Availability

The datasets used and/or analyzed during the current study are available from the corresponding author on reasonable request.
